# Electrochemical Mechanism of the Preparation of High-Purity Indium by Electrodeposition

**DOI:** 10.3389/fchem.2022.871420

**Published:** 2022-05-24

**Authors:** Zhongmin Hou, Xiaomin Wang, Jidong Li, Zhen Li, Yiyong Wang, Hongxuan Xing

**Affiliations:** ^1^ Liaoning Key Laboratory of Chemical Additive Synthesis and Separation, School of Materials Science and Engineering, Yingkou Institute of Technology, Liaoning Yingkou, China; ^2^ School of Materials and Metallurgy, University of Science and Technology Liaoning, Liaoning Anshan, China

**Keywords:** indium, electrodeposition, indium sulfate, cyclic voltammetry, nucleation mechanism

## Abstract

Indium is a crucial material and is widely used in high-tech industries, and electrodeposition is an efficient method to recover rare metal resources. In this work, the electrochemical behavior of In^3+^ was investigated by using different electrochemical methods in electrolytes containing sodium and indium sulfate. Cyclic voltammetry (CV), chronoamperometry (CA), and alternating current impedance (EIS) techniques were used to investigate the reduction reaction of In^3+^ and the electrocrystallization mechanism of indium in the indium sulfate system. The cyclic voltammetry results showed that the electrodeposition process is irreversible. The average charge transfer coefficient *a* of In^3+^ was calculated to be 0.116 from the relationship between the cathodic peak potential and the half-peak potential, and the H^+^ discharge occurred at a higher negative potential of In^3+^. The nucleation mechanism of indium electrodeposition was analyzed by chronoamperometry. The mechanism of indium at potential steps of −0.3 to −0.6 V was close to diffusion-controlled instantaneous nucleation with a diffusion coefficient of 7.31 × 10^−9^ cm^2^ s^−1^. The EIS results demonstrated that the reduction process of In^3+^ is subject to a diffusion-controlled step when pH = 2.5 and the applied potential was −0.5 V. SEM and XRD techniques indicated that the cathodic products deposited on the titanium electrode have excellent cleanliness and purity.

## Introduction

Indium is a bright and silvery-white rare metal with low melting point, high flexibility, high boiling point, and a face-centered tetragonal crystal structure. At room temperature, indium is not oxidized by air and its chemical properties are relatively stable (Katiyar and Randhawa, 2020). With the increasing demand for indium in the high-tech field, the purity of indium has become more demanding. High-purity indium is mainly used in semiconductors, fluorescent materials, ITO films, and photovoltaic solar cells (Xu et al., 2021; Luo et al., 2022). In addition, high purity indium has become the top classification of critical materials due to its wide application, such as liquid crystal displays and light-emitting diodes (Fontana et al., 2015; Rakhymbay et al., 2016).

In order to achieve a better supply of indium in globalization, indium is mainly extracted from zinc ore, sublimate, and lead-containing materials, and the recovery rate is 50–60% (Cheng et al., 2021; Xu et al., 2021). The indium resources of China rank first in the world. In 2006, China’s indium output accounted for more than 60% of the world but it was highly dependent on exports and the supply of indium could not meet the demand. At present, the critical challenge in dealing with the new market is to seek the raw material supply of indium (Kang et al., 2011).

In the extraction and purification of indium, a combination of chemical cleaning and physical purification is mainly adopted, including the electrolytic refining method, float zone smelting method, vacuum distillation method, and metal–organic compound method (Bardi et al., 2009; Liu et al., 2021). Based on hydrometallurgy, electrolytic refining of the aqueous solution is paid more attention because of its advantages of the simple operation process and low cost of equipment. Yu et al. (2013) studied the effects of acidity, current density, and other parameters on electrolytic purification and controlled the optimal electrolytic conditions, which are more conducive to the purification of indium. Liu (2010) used glycerol potassium iodide for the prior removal of Cd and Tl and investigated the effect of acidity on the product purity and current efficiency in indium electrolytic refining; the optimal pH of the electrolytic process was determined to be 2–3.

The electrical crystallization of metals mainly consists of the atomic generation of crystalline nuclei after the ions enter the electrolyte and discharge at the electrode surface. The overpotential has a specific influence on the rate of nuclei formation (Zhao et al., 2014; Shelke et al., 2020; Liu et al., 2021). It is possible to refine crystallization only if the rate of the crystal nucleus is greater than the crystal growth rate. The complexity of the electron crystallization process is related to the inhomogeneity of the crystal surface and the generation of new phases (Eliaz and Sridhar, 2008).

Indium deposition includes chemical deposition and electrodeposition. Electrodeposition is an excellent preparation method and involves low cost, high speed, and extensive film (Zhang et al., 2001; Avchukir et al., 2018). In the early stages of metal deposition, metal ions were transferred by hemispherical diffusion and three-dimensional diffusion. The process of indium deposition is affected by the hydrogen overpotential. Using electrochemical methods, Li et al. (2006) demonstrated the mechanism of the cathodic reaction in indium sulfate type plating and found that the cathodic reduction process involves prechemical and irreversible electrochemical reactions. Ciro et al. (2020) examined the indium electrode process using different metals as cathodes and concluded that Ni and SS are the most suitable cathode carriers. Rakhymbay et al. (2016) studied the deposition mechanism of indium by the electrochemical method and calculated the diffusion coefficient and diffusion activation energy. Pettit et al. (2002) researched the kinetics of indium deposition on molybdenum substrates and concluded that SHG and FFT-EIS can be used to investigate electrodeposition surface modification processes. Luo et al. (2022) analyzed the removal mechanism of lead and the behavior of selenium in the purification process by controlling the potential oxidation and vacuum distillation, indicating that lead impurities can be removed by combining these two methods effectively.

In this work, the electrodeposition process of indium in an indium sulfate system was studied by cyclic voltammetry, chronoamperometry, and the AC impedance method. The function and mechanism of cathode recovery of indium were determined. The deposition layer was scraped off, and the morphology was observed by scanning electron microscopy. The crystallinity of indium in the range of 10°–90° was evaluated using an x-ray energy dispersive spectrometer. The study of the electrochemical behavior of indium ions can lay a foundation for the preparation of high-purity indium and the electrochemical regulation of impurities, which can provide theoretical guidance for the electrolytic refining process of crude indium.

## Materials and Methods

Indium sulfate used in this experiment was provided by Shanghai McLin Biochemical Technology Co., Ltd. The purity was 99.99%, which met the ideal requirements. Anhydrous sodium sulfate was provided by Liaoning Quanrui Reagent Co., Ltd. with the purity of AR. In electrochemistry experiments, indium sulfate was not added in the control experiment. The electrolyte in one group contained only sodium sulfate at a concentration of 0.07 mol/L. The other group of experimental electrolytes contained indium sulfate and sodium sulfate at concentrations of 0.03 mol/L and 0.07 mol/L, respectively. A three-electrode system was used to study the reaction mechanism of the electrode. The effective area of the titanium plate as a working electrode was 9.5 cm^2^, and the effective area of the current timing test was 8.74 cm^2^. The working electrode was polished, cleaned, and soaked in anhydrous ethanol prior to use. The surface of the platinum electrode was carefully polished with 1000 sandpaper, which removed the oxide film and then wiped with alcohol cotton. Finally, it was ultrasonically cleaned with distilled water and anhydrous ethanol and then placed in anhydrous ethanol for 20 min before use. In the electrodeposition experiment, coarse indium was used as an anode and a high-purity titanium plate was used as a cathode (Lobaccaro et al., 2014; Dell'Era et al., 2020). The electrolyte was an indium sulfate electrolytic system, and NaCl was added to enhance its conductivity (Walsh and Gabe, 1981; Liu et al., 2021).

The experimental mechanism is as follows:

Anode:

$$\text{In}-3{\text{e}}^{-}=\text{I}{\text{n}}^{3+}$$ (1)

$$\text{Me}-\text{n}{\text{e}}^{-}=\text{M}{\text{e}}^{\text{n}+}$$ (2)

Cathode:

$$\text{I}{\text{n}}^{3+}+3{\text{e}}^{-}=\text{I}\text{n}$$ (3)

$$\text{M}{\text{e}}^{\text{n}+}+\text{n}{\text{e}}^{-}=\text{Me}$$ (4)

$$\text{2H}+{\text{2e}}^{-}={\text{H}}_{2}$$ (5)

## Results and Discussion

### Cyclic Voltammetry Analysis

**FIGURE 1:**
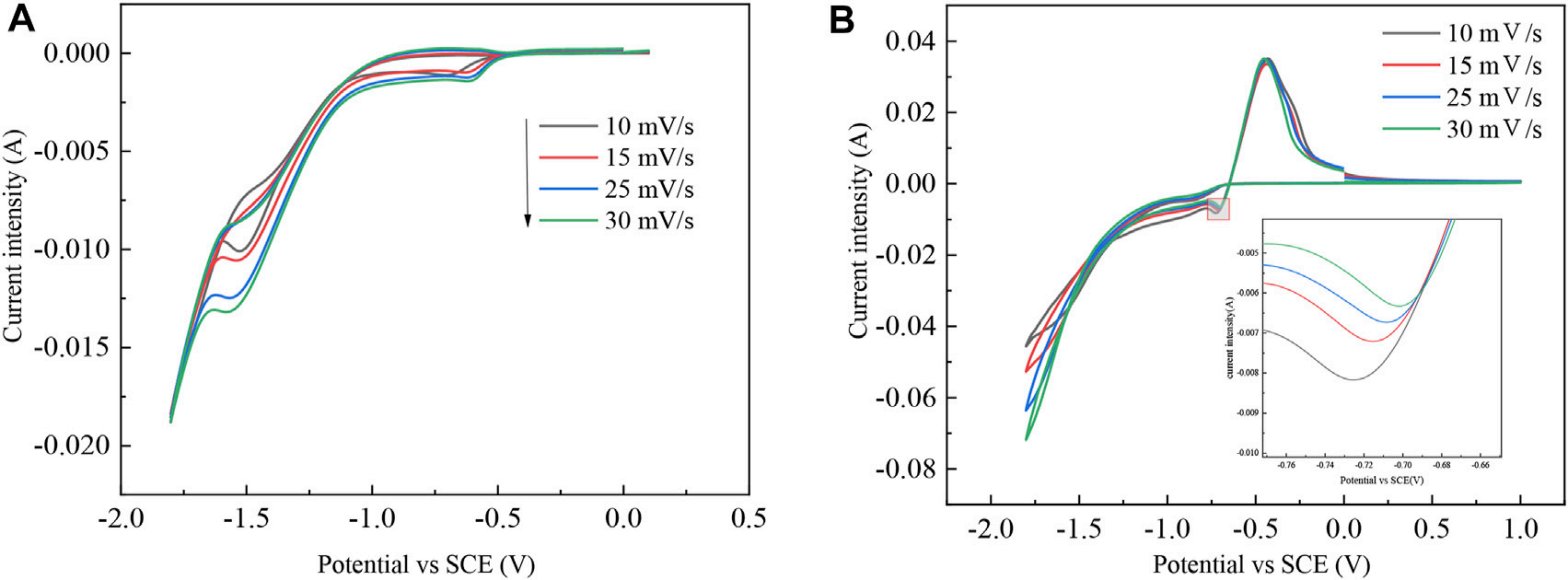
CV curves containing different electrolytes: **(A)** 0.07 mol/L Na_2_SO_4_ and **(B)** 0.03 mol/L In_2_(SO_4_)_3_ and 0.07 mol/L Na_2_SO_4_.

The electrode reaction mechanism on the cathode surface with or without In^3+^ as an electrolyte was determined by cyclic voltammetry. The test temperature was 298 K. The potential was set from 1 V to −1.8 V with pH = 2.5 in the indium sulfate system. The test results are shown in Figure 1.

During the electrolysis of indium dissolved in the anode, the indium in the crude indium enters the electrolyte in an ion form. The redox process of In^3+^ is determined from the preliminary CV curves. In the electrolyte containing sodium sulfate, the hydrogen discharge potential in the Ti metal carrier is approximately −1.06 V, which indicates that the H^+^ discharge occurs only at a higher negative potential of the indium discharge and also verifies that H^+^ has a large overpotential for indium during the electrolytic refining process. The reduction of In^3+^ can be achieved on a titanium plate. There is an oxidation peak and a reduction peak on the curve, where it can be assumed that indium in crude indium enters the electrolyte as an ion and is oxidized to In^3+^. Under the action of electric field forces, In^3+^ migrates from the anodic region to the cathodic area, where it is reduced to In by getting three electrons at the cathode. Therefore, indium is diminished by an irreversible reaction of three electrons in one step, with a reduction potential between −0.65 V and −0.72 V and an oxidation potential between −0.42 V and −0.45 V (Rakhymbay et al., 2016; Liu et al., 2021).

The cross potential shows nucleation of In, which lays a foundation for further study of the nucleation mechanism. As shown in the inset of Figure 1B, the reduction peak shifts positively as the sweep speed increases.

To further prove the number of electrons transferred during the reduction of In^3+^ on the titanium plate, the relevant data were calculated and tabulated at any four points on the right-hand side of the cyclic voltammetric curve, using 30 mV/s as an example. This is shown in Table 1.

**TABLE 1:** Calculated data related to any four points in the cyclic voltammetric curve (30 mV/s).

*I*_*p*_/mA	*I*/mA	*E*/V	*lg[(I*_*p*_ *-I)/I*]
−6.34	−6.31	−0.923	−2.323
−6.34	−6.29	−0.920	−2.09
−6.34	−6.27	−0.918	−1.959
−6.34	−6.25	−0.916	−1.842

**FIGURE 2:**
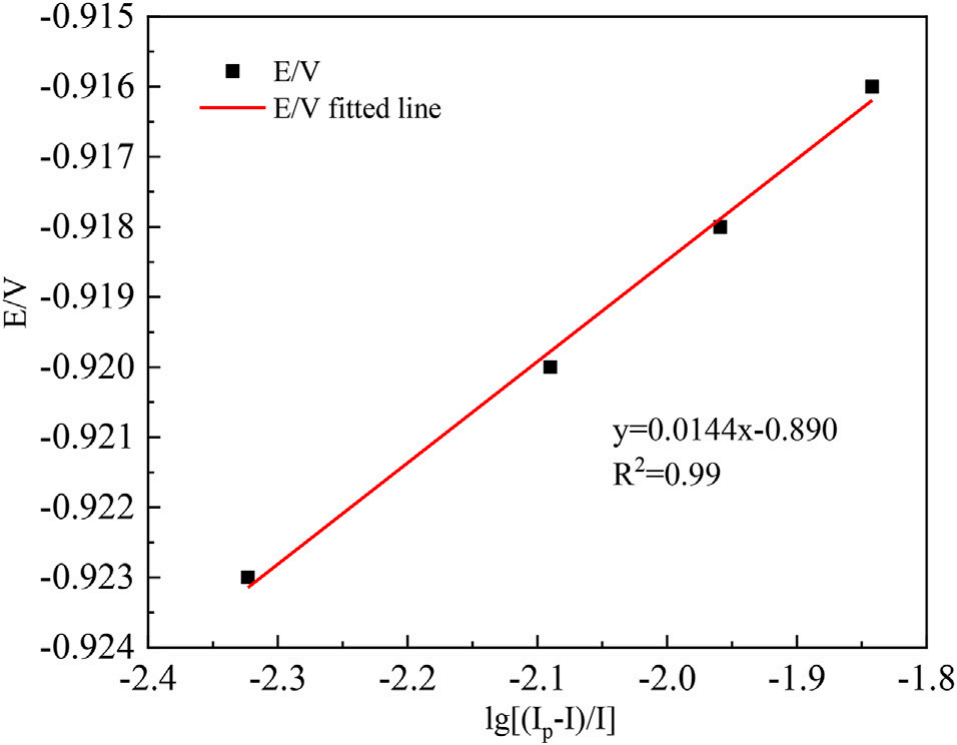
Graph of the *E*–lg [(*I*_p_-*I*)/*I*] relationship (30 mV/s).

Based on the relevant data in this table, the *E*–lg[(*I*_p_-*I*)/*I*] relationship is plotted as shown in Figure 2.

Figure 2 shows that the E-lg[(I_p_-I)/I] curve shows a good linear relationship with a fitted straight line slope *K* = 0.0144. According to the electron transfer number equation (Li et al., 2019), the number of electrons transferred during In^3+^ reduction can be calculated as follows:

$$E=Y+\frac{1.857RTlg\left[\left(I_{p}-I\right)/I\right]}{nF}$$ (6)

The slope $K=\frac{1.857RT}{nF}=\text{0}\text{.}0144$ . It follows that the reduction peak of the cyclic voltammetric curve at 30 mV/s corresponds to the number of electrons transferred by In^3+^ at the Ti electrode:

$$n=\frac{1.857\times 8.314\times 297}{96500\times 0.0144}=3.30\approx 3$$ (7)

In order to study further the reversibility and charge transfer coefficient of the reaction of indium in crude indium on the titanium electrode, the test data in Figure 1B are drawn as shown in Table 2.

**TABLE 2:** Parameters corresponding to cyclic voltammetry curve of In^3+^ on Ti electrode at 298 K.

*v*/V·s^−1^	0.010	0.015	0.025	0.030
*lnv/ln(V/s)*	−4.605	−4.200	−3.689	−3.507
*v* ^*1/2*^ */(V/s)* ^*1/2*^	0.100	0.122	0.158	0.173
*E*_*pc*_/V	−0.723	−0.715	−0.703	−0.701
*−I*_*pc*_/mA	8.14	7.21	6.67	6.33
*−E*_*pa*_/V	0.432	0.437	0.442	0.450
*I*_*pa*_/mA	35.13	33.59	34.8	35.11
*E*_*p/2*_/V	−0.578	−0.576	−0.573	−0.576
∣*E*_*pc*_ *−E*_*p/2*_∣/V	0.145	0.139	0.130	0.125
*α*	0.101	0.114	0.122	0.127

**FIGURE 3:**
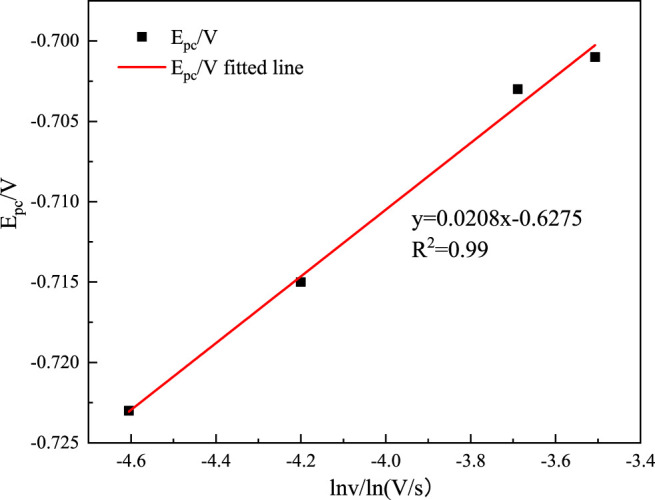
Relationship between *E*_pc_ and ln*v*.

According to the data in Table 2, the reduction peak potential *E*_*p*_ and the logarithmic of the scanning speed ln *v* are plotted, as shown in Figure 3. As shown in Figure 3, the potential (*E*_*p*_) linearly relates well the natural logarithm of the scan rate and further proves that the reduction process of In^3+^ at the Ti electrode is irreversible. Its peak potential *E*_*pc*_ and half-peak potential *E*_*p/2*_ satisfy the following equation (Xiao et al., 2017):

$$|E_{pc}-E_{p/2}|=\frac{1.857\text{R}\text{T}}{\alpha nF}$$ (8)

where *E*_*pc*_ is the cathodic peak potential (V); *E*_*p/2*_ is the half-peak potential (V); *α* is the charge transfer coefficient; *n* is the number of electrons transferred; *F* is the Faraday constant (C mol^−1^), *R* is the gas constant (J (mol K)^−1^); and *T* is the absolute temperature(K). Substituting the relative data into Equation 8, the average value of the charge transfer coefficient can be calculated to be 0.116.

**FIGURE 4:**
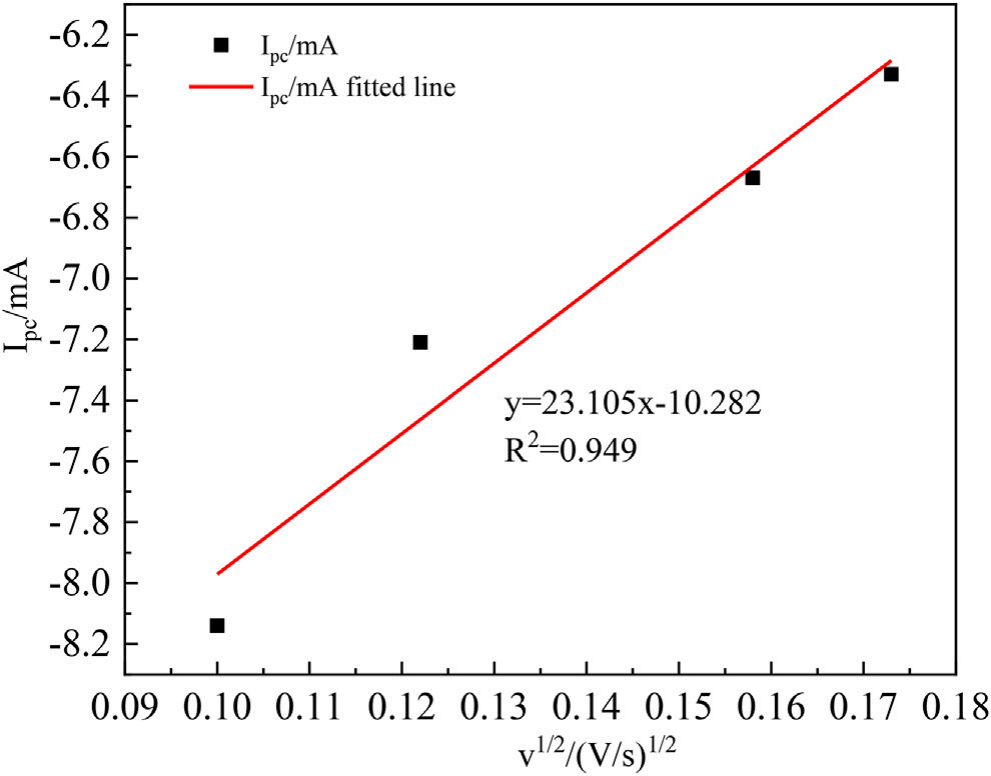
*v* ^*1/2*^ relationship with the peak current *I*_*pc*_.

The square root of the scanning speed *v* ^*1/2*^ versus the reduction peak current *I*_*pc*_ is shown in Figure 4. The controlling steps of the electrochemical reaction of metal ions in an electrolyte solution can be determined assuming that a wonderful proportion relationship is found after fitting. In that case, the diffusion coefficient of In^3+^ in the system can be calculated according to the Berzins–Delahay equation (Kang et al., 2020; Li et al., 2020).

Figure 4 shows that *I*_*pc*_ and *v* ^*1/2*^ have an excellent linear relationship, indicating that the electrochemical reduction process of In^3+^ is controlled by the diffusion control step, and the diffusion coefficient of In^3+^ can be found from the Berzins–Delahay equation (Li et al., 2019; Rajska et al., 2021):

$$I_{pc}=\;0.496nFSC_{0}{\left(\frac{\alpha nvFD_{In^{3+}}}{RT}\right)}^{\frac{1}{2}}$$ (9)

where *I*_*pc*_ is the cathodic peak current; *n* is the number of electrons transferred; *F* is the Faraday constant (C mol^−1^), *R* is the gas constant (J (mol K)^−1^); *T*is the absolute temperature (K); *α* is the charge transfer coefficient; *S* is the working electrode area (cm^2^); *C*_*0*_ is the In^3+^ concentration (mol L^−1^); *v* is the sweep velocity (mV s^−1^); $D_{In^{3+}}$ is the diffusion coefficient of In^3+^(cm^2^ s^−1^); and *n* is the number of electrons transferred.

According to Eq. (9), when *I*_*p*_ */v* ^*1/2*^ = 23.105 mA s mV^−1^, *n* = 3, *F* = 96,485 C mol^−1^, *R* = 8.314 J (mol K)^−1^, *T* = 298 K, *α* = 0.116, *S* = 9.5 cm^2^, *C*_*0*_ = 0.06 mol cm^−3^, it can be computed that $D_{In^{3+}}=5.88\times 10^{-9}cm^{2}\cdot s^{-1}$

### Chronoamperometric Analysis

**FIGURE 5:**
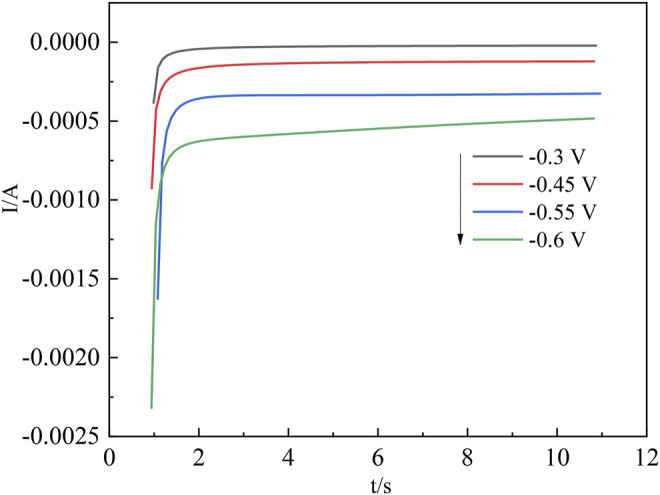
Chronoamperometric current curves for In^3+^ at different potentials.

**FIGURE 6:**
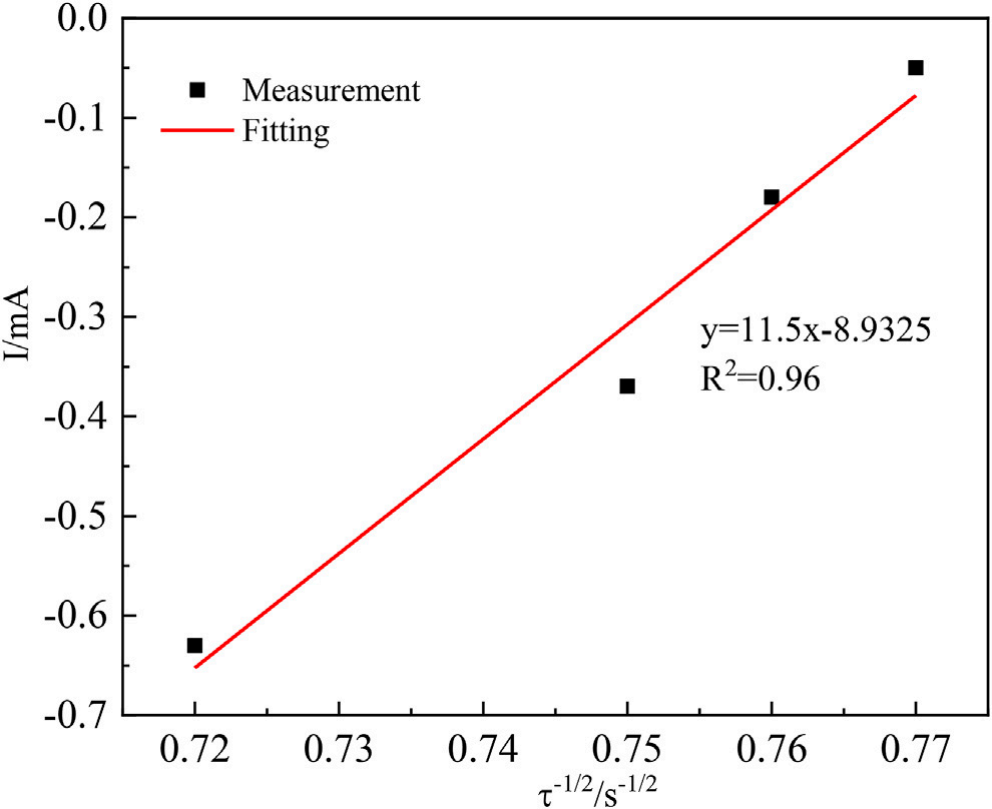
Relationship between the current intensity *I(t)* and the negative square root of the transition time (*τ* ^*−1/2*^).

The nucleation process in acidic aqueous solutions of indium sulfate containing sodium sulfate was studied by chronoamperometry. The temperature was 298 K and the potential ranged from −0.3 V to −0.6 V. The experimental results are shown in Figure 5. The relationship between current intensity and the negative square root of the transition time is shown in Figure 6.

The diffusion coefficient of In^3+^ was calculated from the Sand equation (Kang et al., 2020) as follows:

$$I\tau^{\frac{1}{2}}=\;\frac{nFC_{o}S{\left(\pi D_{In^{3+}}\right)}^{\frac{1}{2}}}{2}$$ (10)

The transition time τ^−1/2^ in the timing current shows a good linear relationship with the current intensity, further demonstrating that the diffusion step controls the electrochemical reduction of In^3+^ on the cathode surface (Ciro et al., 2021). In the irreversible system, the diffusion coefficient of In^3+^ can be calculated according to Equation 10, where *S* is the working electrode surface area (cm^2^); $D_{In^{3+}}$ is the diffusion coefficient (cm^2^/s); *C*_*0*_ is the In^3+^ concentration (mol L^−1^); *τ* is the transition time; π = 3.14; *C*_*0*_ is the In^3+^ concentration (mol L^−1^); *F* = 96,485 C mol^−1^; and *n* is the number of electrons transferred.

When *S* = 8.74 cm^2^, *Iτ* ^1/2^ = 11.5 mA s, *F* = 96,485 C mol^−1^, *C*_*0*_ = 0.06 mol L^−1^, *n* = 3, and π = 3.14, it can be computed that $D_{In^{3+}}=7.31\times 10^{-9}cm^{2}\cdot s^{-1}$ .

Due to the difference in experimental conditions, the calculated results are close to those of other studies (Rakhymbay et al., 2016; Ciro et al., 2021). The process by which In^3+^ is reduced at the Ti electrode surface to form indium nuclei can be derived from the current timing curves at different potentials (Chung and Lee, 2013). The nucleation mechanism of In^3+^ can be obtained from this process by fitting the equations for instantaneous nucleation and continuous nucleation (Katiyar and Randhawa, 2020; Xu et al., 2021) to four different points taken arbitrarily from the ascending parts of the different timing current curves in Figure 5, respectively.

Instantaneous nucleation:

$$I\left(t\right)=ZFN\pi {\left(2D_{In^{3+}}C_{0}\right)}^{\frac{3}{2}}M^{\frac{1}{2}}\rho^{-\frac{1}{2}}t^{\frac{1}{2}}$$ (11)

Continuous nucleation:

$$I\left(t\right)=\frac{2}{3}ZFK_{n}N\pi {\left(2D_{In^{3+}}C_{0}\right)}^{\frac{3}{2}}M^{\frac{1}{2}}\rho^{-\frac{1}{2}}t^{\frac{3}{2}}$$ (12)

In the above equations, *I(t)* is the current intensity corresponding to time (mA); *Z* is the valence; *F* = 96,485 C mol^−1^; *N* is the nucleation number density (cm^−2^); $D_{I{\text{n}}^{3+}}$ is the diffusion coefficient(cm^2^ s^−1^); *C*_*0*_ is the concentration of In^3+^ (mol L^−1^); *M* is the molar mass of the sediment (g mol^−1^); *ρ* is the density of the sediment (g cm^−3^); *K*_*n*_ is the nucleation rate constant (cm^−2^s^−1^); *t* is the time (s); and π = 3.14.

The above equations can be simplified as shown in Equations 13 and 14, respectively.

Instantaneous nucleation:

$$I\left(t\right)=kt^{\frac{1}{2}}+b_{1}$$ (13)

Continuous nucleation:

$$I\left(t\right)=kt^{\frac{3}{2}}+b_{2}$$ (14)

**FIGURE 7:**
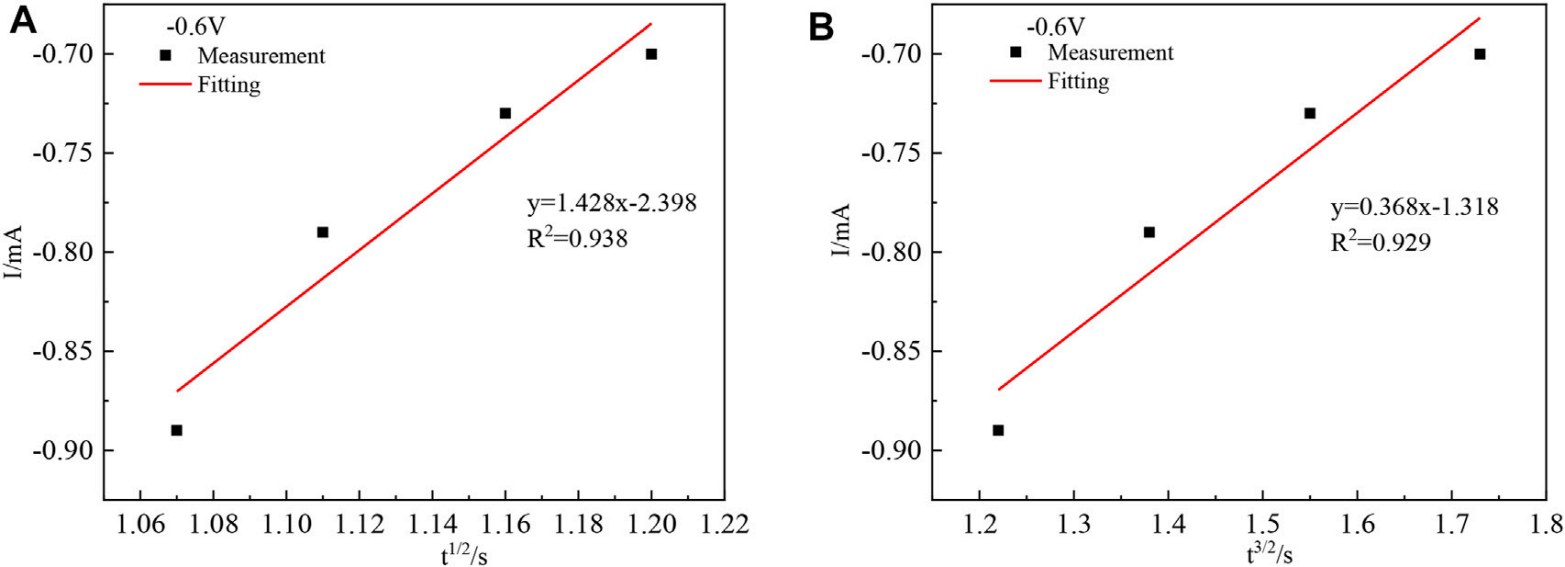
Relationship between *I*(*t*)-*t* ^1/2^ **(A)** and *I*(*t*)-*t* ^3/2^ **(B)**.

The *I(t)–t* ^*1/2*^ and *I(t) –t* ^*2/3*^ relationships were plotted, and linear fits were made to *I(t)–t* ^*1/2*^ and *I(t) –t* ^*2/3*^, respectively, to determine the nucleation mechanism of In. Taking any four points from the ascending part of its current timing curve at −0.6 V, the *I(t) –t* relationship is plotted as shown in Figure 7A and Figure 7B.

Figure 7 shows that the goodness of fit of the fitted line (A) is greater than that of the fitted line (B), and the chronoamperometric plot is linear with the square root of time *t* ^*1/2*^, indicating that the nucleation mechanism of indium on the titanium plate is instantaneous nucleation.

### Indium Electrowinning

**FIGURE 8:**
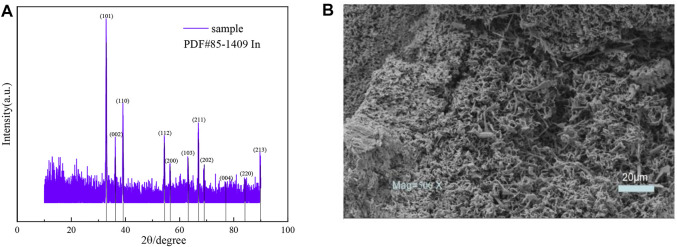
**(A)** XRD patterns and **(B)** SEM image of indium deposited on Ti electrodes.

The cathode products were characterized by XRD and SEM techniques during the electrodeposition of Ti as a metal carrier. The test results are shown in Figure 8.

A distinct diffraction peak for indium at 32.94° indicates a relatively pure product with good crystallinity. The Ti plating shows irregular grain morphology. During the deposition of indium with the Ti electrode, the microscopic images show uneven deposition and porous open holes with more pronounced dendrite growth, which indicates a high roughness of the cathode.

### AC Impedance Analysis.

**FIGURE 9:**
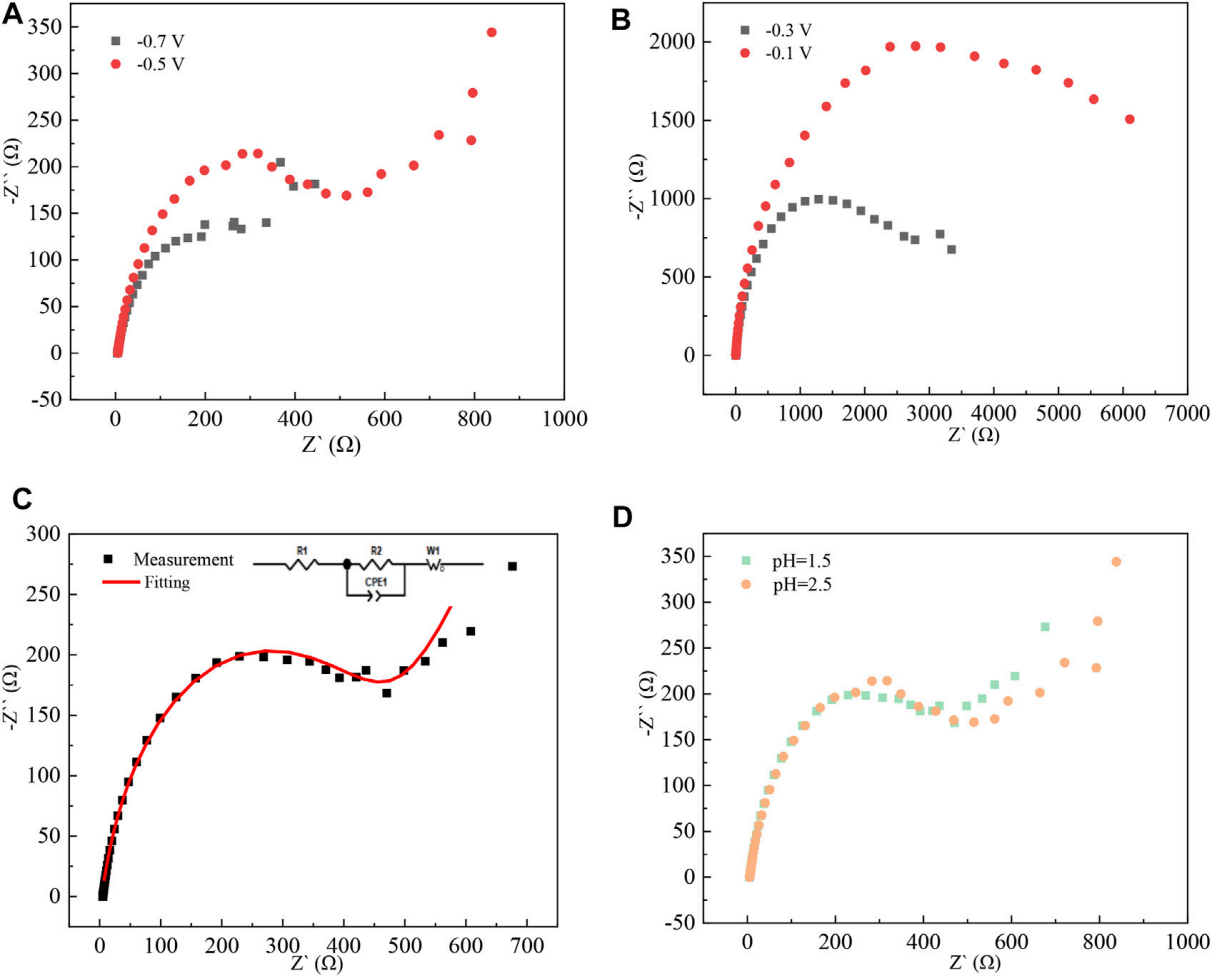
Nyquist pattern of high-purity indium prepared by electrodeposition at different pH values: **(A)** pH = 2.5, potential −0.7 V, −0.5 V; **(B)** pH = 1.5, potential −0.3 V, −0.1 V; **(C)** pH = 1.5, potential −0.5 V; and **(D)** potential of −0.5 V, pH = 1.5, 2.5

The electrochemical reaction in the cathodic process was further studied by the AC impedance method (Pettit et al., 2002). The test frequency was 5 × 10^−3^–1 × 10^5^ Hz, and the test temperature was 298 K. Z-view software is used to fit the data to obtain the corresponding equivalent circuit diagrams. Ac impedance test results are shown in Figure 9.

When the pH is 1.5 and the applied potential is −0.3 V and −0.1 V, there is only a capacitive arc with a large radius, indicating that the deposition of indium on the titanium plate in the indium sulfate electrolytic liquid system is an electrochemically controlled step. As the applied potential increases, the radius of the capacitive arc decreases, indicating that the deposition layer formed at the high potential is complete and that the resistance of the electrochemical reaction that occurs at this time is reduced. When the applied potential is −0.5 V and pH = 2.5, a straight line appears in the low-frequency region, indicating that the electrochemical reduction process of In^3+^ is subject to a diffusion-controlled step, which is consistent with the results of cyclic voltammetry tests.

## Conclusion

The electrochemical behavior of In^3+^ was investigated by using different electrochemical methods in electrolytes containing sodium and indium sulfate. The reduction mechanism of In^3+^ on titanium plates is as follows: In^3+^+3e^−^→In, and the electrochemical reduction process is irreversible and controlled by a diffusion step. The average diffusion coefficient of In^3+^ is $D_{I{\text{n}}^{3+}}=6.595\times 10^{-9}cm^{2}\cdot s^{-1}$ . The CA results show that the deposition of indium on titanium cathodes is a transient nucleation process. The SEM morphology shows that indium is porous and highly crystalline at the titanium electrode. Titanium electrodes are a suitable cathode substrate for the electrodeposition of indium and they can be used for the optimization of high-purity indium processes in electrolytic refining.

## Data Availability

The original contributions presented in the study are included in the article/Supplementary Material, further inquiries can be directed to the corresponding author.
